# Maternal serum lipids in some women with pre-eclampsia in Yaoundé

**DOI:** 10.11604/pamj.2021.39.14.22734

**Published:** 2021-05-05

**Authors:** Jean-Thierry Ebogo-Belobo, Céline Mimboe Bilongo, Roger Ahouga Voufo, Efietngab Atembeh-Noura, Ousmanou Djabidatou, Martial Tsague Kenfack, Therese Pulcherie Ateba, Patrice Enoka

**Affiliations:** 1 Institute of Medical Research and Medicinal Plant Study, Yaoundé, Cameroon,; 2 Catholic Hospital “Mgr. Jean Zoa” of Nkolndongo, Yaoundé, Cameroon,; 3 School of Health Sciences, Catholic University of Central Africa, Yaoundé, Cameroon,; 4 School of Laboratory Technicians, Yaoundé, Cameroon

**Keywords:** Serum lipid profile, preeclampsia, high blood pressure

## Abstract

Preeclampsia is one of the most common complications of pregnancy and occurs in approximately 3-8% of all pregnancies worldwide. Although the aetiology of preeclampsia still largely remains unclear, it is thought to be related to endothelial dysfunction and can lead to serum lipid abnormalities. Therefore, this case-control study was conceived and designed with the aim to compare maternal lipid profile parameters and cardiovascular risk factors, between preeclamptic and healthy pregnancies. Blood samples were collected after overnight fasting from 48 preeclamptics and 96 healthy pregnant controls matched for age and gestational weeks and serum lipid profile concentrations were estimated and used them to calculate cardiac risk ratio I and II. There was a significant rise in serum lipid levels in pregnancies complicated by preeclampsia. These lipids turn out to be risk factor for cardiovascular diseases. Positive correlation of maternal serum lipids to high blood pressure suggests a causal relationship.

## Introduction

Preeclampsia is one of the most common complications of pregnancy and occurs in approximately 3-8% of all pregnancies worldwide [1]. It is a pregnancy-associated condition unique to humans characterized by an onset of hypertension and proteinuria. It occurs after 20 weeks of gestation and increases the risk of maternal mortality, as well as neonatal morbidity and/or mortality [1,2]. Even if, the etiology of preeclampsia still remains unclear, it is thought to be related to endothelial dysfunction which causes placental ischemia and maternal syndrome (oxidative stress and a generalized inflammatory state) [3]. This failure in endothelial remodeling, can lead to maternal serum lipid abnormalities due to placental oxidative stress [3]. Several studies have shown that dyslipidemia might be associated with risk of preeclampsia. Thus, elevated serum Total Cholesterol (TC) levels, Low Density Lipoprotein cholesterol (LDL-c) level, Very Low Density Lipoprotein (VLDL), levels and Triglycerides (TG) levels have been found in women with preeclampsia as compared to normotensive pregnant women [4,5]. Otherwise, an abnormal lipid profile is known to be strongly associated with atherosclerotic cardiovascular diseases [6] and has a direct effect on endothelial dysfunction. Evidence has suggested that pregnancies accompanied by preeclampsia are associated with an increased risk of cardiovascular disease and metabolic syndrome [6-8]. Although the modification of lipid metabolism in normal and preeclamptic pregnancies has been widely studied elsewhere, data on this important topic and particularly on link with cardiovascular diseases risk in Cameroon are scarce. Therefore, the present study was conceived and designed with the aim to compare maternal lipid profile parameters and cardiovascular risk factors, between preeclamptic and healthy pregnancies.

## Methods

**Study design/study site:** this case-control study was carried out from May 2018 to January 2019, at the Catholic Hospital “Mgr. Jean Zoa” of Nkolndongo/Yaoundé Cameroon.

**Study population:** pregnant women with gestational age greater than or equal to 20 weeks, with an elevated blood pressure (diastolic blood pressure (DBP) of ≥90 mmHg and/or systolic blood pressure (SBP) ≥140 mmHg) associated with proteinuria of at least 1+ on dipstick examination were recruited as cases (preeclamptic group). Normotensive pregnant women with gestational age greater than or equal to 20 weeks, matched for age and gestational age with the cases and receiving antenatal care at the maternity unit were recruited as controls (controls group). All eligible cases were recruited using a consecutive sampling method and each case was matched with two controls. Only pregnant women who presented to the clinic in a fasting state (at least 8 hours) and who gave their consent were included. A thorough history and physical examination was done by the Physician and women with other hypertensive disorders of pregnancy such as eclampsia or chronic hypertension, and other conditions such as diabetes mellitus, autoimmune disease, renal disease or intake of drugs which affect lipid metabolism were excluded.

**Ethical considerations:** the study was approved by the Centre Regional Ethics Committee for Human Health Research, no/0440/CRERSHC/2018 and permission to recruit participants was obtained from the administrative department of the hospital. Informed consent was obtained from all participants before enrollment into the study. Privacy and confidentiality conditions were respected by de-linking all samples from personal identifiers and assigning unique code during recruitment.

**Blood pressure measurement:** the blood pressure was measured by trained nurses using a mercury sphygmomanometer and a stethoscope by palpatory and auscultatory methods. The procedure was repeated for each patient. Mean values of duplicate measurements were recorded as the blood pressure.

**Biochemical analysis:** a volume of 5 ml of venous blood samples were taken after overnight fasting from all the participants. The sample was centrifuged and the serum was taken to determine lipid profile level (total cholesterol (TC); triglyceride (TG); HDL-cholesterol (HDL-c) by enzymatic techniques using cypress diagnostic kits and LDL-cholesterol (LDL-c) was calculated using Friedewald´s formula. Cardiac risk ratio I (CRR I) and Cardiac risk ratio II (CRR II) were calculated by using the values of lipid profile parameters in the following way:

$$\text{CRR I}=\frac{TC}{HDL-c}\text{ and CRR II=}\frac{LDL-c}{HDL-c}$$

**Statistical analysis:** statistical Package for Social Sciences (SPSS) version 23 for Windows (IBM Corp. Armonk, NY, USA) was used for data analysis. Quantitative variables (age, BMI, blood pressure, lipid profile parameters, CRR I and II) were expressed as mean and standard deviation (X ± SD). Independent student´s t test was used for comparison of means of clinical and biochemical parameters between 2 groups of participants. Correlations between clinical and biochemical parameters of participants were estimated by Pearson´s correlation. The odds ratio of the association between preeclampsia and cardiovascular risk were performed using Chi-square test. The results were considered significant when the p-value was less than 0.05.

## Results

A total of 144 pregnant women comprising 48 preeclamptics and 96 healthy normotensive controls were recruited using a consecutive sampling method. The case and control groups were not statistically different in age and weeks of gestation (Table 1). Body mass index (BMI), which was recorded at the time of blood sampling was not significantly different (P = 0.064) between two groups. The mean diastolic and systolic blood pressures, cardiac risk ratio I and cardiac risk ratio II of the preeclamptics were significantly higher than in normotensive women (Table 1). Increase of lipid profile parameters was significant in the case group for Total Cholesterol (TC) and LDL-c. No significant differences were observed in other measured lipid profile parameters (Table 1). The results of Pearson´s correlation show that DBP positively correlated with TC (r = 0.468, P < 0.01), LDL-c (r = 0.501, P < 0.01), CRR I (r = 0.327, P < 0.01) and CRR II (r = 0.423, P < 0.01). SBP positively correlated with TC (r = 0.368, P < 0.01), LDL-c (r=0.465, P< 0.01), CRR I (r = 0.276, P < 0.01) and CRR II (r = 0.368, P < 0.01). A similar relationship was observed between BMI and TC (r = 0.308, P< 0.01), BMI and TG (r = 0.494 P < 0.01) (Table 2). Table 3 presents evaluation of cardiovascular risk. The cardiac risk was significantly higher in women with preeclampsia than in normotensive women. For CRR I ˃5, the risk was 7.86 (95% CI, 3.12-19.79; P ˂0.001) and for CRR 2 ˃3, the risk was 5.50 (95% CI, 2.15-14.08, P ˂0.001) (Table 3).

**Table 1:** clinical and biochemical characteristic of the participants

Variables	Preeclamptic (n=48)	Control (n=96)	p-value
Age (years)	28.83±4.10	28.92±4.35	0.912
Gestational age (weeks)	26.92±5.18	26.46±4.75	0.597
BMI (Kg/m^2^)	31.51±7.34	29.60±4.77	0.064
Diastolic blood pressure (mm Hg)	100.00±12.68	64.48±8.85	˂0.001
Systolic blood pressure (mm Hg)	151.42±10.77	105.56±11.10	˂0.001
Total cholesterol(g/L)	1.81±0.40	1.47±0.40	˂0.001
HDL-c(g/L)	0.50±0.15	0.52±0.16	0.346
LDL-c (g/L)	1.14±0.42	0.69±0.46	˂0.001
Triglycerides (g/L)	1.27±0.54	1.36±0.52	0.328
Cardiac risk ratio I (TC/HDL-c)	3.93±1.38	3.10±1.58	0.002
Cardiac risk ratio II (LDL-c/HDL-c)	2.54±1.24	1.52±1.36	˂0.001

BMI: Body Mass Index; LDL-c: Low-density lipoprotein- cholesterol, HDL-c: High-density lipoprotein- cholesterol.

**Table 2:** correlation of clinical, and biochemical parameters of participants

		TC	HDL-c	LDL-c	TG	CRR I	CRR II
**Gestational age**	r	-0.009	0.361**	-0.034	0.042	-0.244**	-0.189*
	p-value	0.916	˂0.01	0,69	0.619	0.003	0,023
**DBP**	r	0.468**	-0.034	0.501**	-0.068	0.327**	0.423**
	p-value	˂0.01	0.684	˂0.01	0.418	˂0.01	˂0.01
**SBP**	r	0.368**	-0.131	0.465**	-0.145	0.276**	0.368**
	p-value	˂0.01	0.118	˂0.01	0.082	˂0.01	˂0.01
**BMI**	r	0.308**	0.082	0.148	0.494**	0.069	0.030
	p-value	˂0.01	0.331	0.077	˂0.01	0.414	0.723

* Correlation=Statistically significant at P<0.05, **Correlation=Statistically significant at P<0.01, r=Correlation coefficient. TC: Total cholesterol, TG: Triglycerides, SBP: Systolic blood pressure, DBP: Diastolic blood pressure, LDL-c: Low-density lipoprotein- cholesterol, HDL-c: High-density lipoprotein-cholesterol, CRR: Cardiac Risk Ratio.

**Table 3:** evaluation of cardiovascular risk

Variable	Preeclamptic (%)	Control (%)	Odds ratio	95% CI	p-value
CRR I					
˃5	20 (41.7)	8 (8.3)	7.86	3.12-19.79	˂0.001
≤5	28 (58.3)	88 (91.7)			
CRR II					
˃3.3	16 (33.3)	8 (8.3)	5.50	2.15-14.08	˂0.001
≤3.3	32 (66.7)	88 (91.7)			

CRR: Cardiac Risk Ratio.

## Discussion

Lipid profile parameters levels have been consistently reported to be elevated in preeclamptic than in normotensive pregnant women [6,7]. These changes may contribute to subsequent risk of abnormalities related to cardiovascular diseases. In this study, results show that some of the lipid fractions (TC and LDL-c) were significantly higher in preeclamptic than normotensive pregnant women. The higher levels of triglycerides along with increase in TC and LDL-c observed in other studies is not consistent with our study. Few studies like those of Chalas *et al*. [9] and Demir *et al*. [10], obtained similar results. To explain this result, Chalas *et al*. [9] suggested that in preeclamptic women, hypertriglyceridemia due to lipoprotein lipase activity, may be inhibited by high Apo C3 concentration. Otherwise, the mean gestational age about 26 weeks may also explain the lack of significant augmentation of triglycerides, as lipid components increase progressively during pregnancy. The increase of lipid component in preeclampsia depends also of dietary habits [11,12].

Our results from Pearson´s correlation analysis, show a significant positive correlation between some lipid parameters (LDL-c and TC) and blood pressure. Correlations noted in this study are in agreement with the reports of previous authors [7] and confirmed suggestions that lipid parameters may be involved in the endothelial damage associated with the pathogenesis of preeclampsia. Endothelial dysfunction, mostly associated with oxidation of LDL-c, leads to the formation of glomerular lesions and subsequently proteinuria, which is associated with preeclampsia as well as give an indication of its severity. Studies have shown consistently that women with a history of preeclampsia have an approximately 2 to 6-fold increased risk to develop future cardiovascular disease [6,13]. In this study, the result appears to suggest an increased cardiovascular risk in preeclamptic women 5-7 times more than in women without preeclampsia. These results support previous literature regarding increased risk of cardiovascular diseases in preeclamptic women. Our study has some limitations among which, the smaller number of recruited pregnant women with pre-eclampsia, which might have affected the power of the study. Although, to overcome this we recruited twice more control subjects than the test subjects in order to maximize the power of the study. As another limit, this study did not take into account the potential confounding factors that may influence the results obtained and our findings should be interpreted with caution, however our results remain similar to those obtained in other African countries [4].

## Conclusion

Serum lipid levels are elevated in pregnancies complicated by preeclampsia, and this may serve as a marker for early diagnosis of the disease. The increase level of lipid profile parameters turns out to be an increased risk factor for future cardiovascular complications.

### What is known about this topic

- Abnormal lipid levels are observed during pregnancy and can lead to preeclampsia which can turn out to be risk factor for cardiovascular diseases;
- Women with a history of hypertensive disorders in pregnancy, and particularly women with recurrent pregnancy disorders, should be candidates for intervention intended to prevent premature cardiovascular disease.

### What this study adds

- In this manuscript, we show that serum lipid levels are elevated in pregnancies complicated by preeclampsia in our study population;
- Positive correlation of maternal serum lipids to high blood pressure suggests a causal relationship like seen in others studies.
